# Supplementary material for: Efficacy of Kinematic Parameters for Assessment of Temporomandibular Joint Function and Disfunction: A Systematic Review and Meta-Analysis
Source: Bioengineering (Basel). 2022 Jun 22;9(7):269. doi: 10.3390/bioengineering9070269 (PMC9311583; doi:10.3390/bioengineering9070269)
Supplement: Supplementary file 1 [file bioengineering-09-00269-s001.zip › bioengineering-1700156-supplementary.pdf]

bioengineering

MDPI

Supplementary Materials

Efficacy of Kinematic Parameters for Assessment of Temporomandibular Joint Function and Disfunction: A Systematic Review and Meta-Analysis

Alessandra Scolaro [1,2], Shahnawaz Khijmatgar [1,2], Pooja Mali Rai [1,2], Francesca Falsarone [2], Francesca Alicchio [2], Arianna Mosca [2], Christian Greco [3], Massimo Del Fabbro [1,4] and Gianluca Martino Tartaglia [1,2,*]

1 Department of Biomedical, Surgical and Dental Sciences, University of Milan, 20122 Milan, Italy; scolaro.al@gmail.com (A.S.); khijmatgar@gmail.com (S.K.); poojarai93@gmail.com (P.M.R.); massimo.delfabbro@unimi.it (M.D.F.)
2 Fondazione IRCCS Cà Granda, Ospedale Maggiore Policlinico, 20122 Milan, Italy; francescafalsarone7@gmail.com (F.F.); f.alicchio@gmail.com (F.A.); ari.mosca97@gmail.com (A.M.); gianluca.tartaglia@unimi.it (G.M.T.)
3 Head of the Unit of Dentistry, Azienda Sanitaria dell'Alto Adige, Merano Hospital, 39100 Bolzano, Italy; christian.greco@sabes.it
4 IRCC Orthopaedic Institute Galeazzi, 20161 Milan, Italy
* Correspondence: gianluca.tartaglia@unimi.it

Table S1. Characteristic Table.

Study	Year	Country	Sample/Sex	Age	Outcomes	Study design	Sponsorship	Diagnostic tool
Bernhard et al. [1]	2003	GER	30 M:19 F:11	21 to 26	Condylar trajectories	Case series	NR	Axhiography
Ghodsi et al. [2]	2018	IR	50 M:33 F:17	26	Condylar trajectories	Case series	NR	Axhiography
Hirai et al. [3]	2009	JAP	22 M:NR F:NR	25 to 68	Incisal trajectories	Case control study	NR	Axhiography
Hue et al. [4]	2011	FRA	60 M: 26 F:34	58 to 64	Condylar angle	Case series study	NR	Axhiography
Sadat Koshari et al. [5]	2003	GER	30 M:11 F:19	Test: 21 ± 7 Control: 17.9 ÷ 7.2	Condylar trajectories	Case control study	NR	Axhiography
Sadat Koshari et al. [6]	2003	GER	8 M:4 F:4	21.8 ± 8.0	Helical axis trajectories	Case series	NR	Axhiography
Tamaki et al. [7]	1997	JAP	45 M:10 F:35	13 to 62	Condylar trajectories, hinge axis	Case series	NR	Axhiography
Wieckiewicz et al. [8]	2014	POL	35 M:0 F:35	10 to 35	Condylar path inclination	Case series	NR	Axhiography

Baqaien et al. [9]	2007	GER	213 M:103 F:110	6.5 to 12.9	Condylar path inclination	Cohort study	NR	Ultrasonic tracking
Kiseri et al. [10]	2018	TUR	35 M.13 F:22	7 to 11	Condylar trajectories	Case series	Scientific Research Projects Coordination Unit of Istanbul University	Ultrasonic tracking
Ko et al. [11]	2012	JAP	50 M:16 F:34	Test: 25.6 ± 3.7 Control: 25.7 ± 5.1	Condylar trajectories	Case control study	NR	Ultrasonic tracking
Ko et al. [12]	2015	JAP	21 M:7 F:14	25.5 ± 4.7	Condylar trajectories, incisal trajectories	Cohort study	NR	Ultrasonic tracking
Kordab et al. [13]	2014	GER	259 M:100 F:159	NR	Condylar path inclination	Case series	NR	Ultrasonic tracking
Linsen et al. [14]	2016	GER	36 M:8 F:28	28.03 ± 6.58	Condylar trajectories, incisal trajectories	Case series	the Department of Prosthodontics, Preclinical Education and Dental Material Science, University Hospital Bonn.	Ultrasonic tracking
Linsen et al. [15]	2021	GER	39 M:15 F.24	48.9 ± 16.2	Condylar trajectories, incisal trajectories	Cohort study	NR	Ultrasonic tracking
Ratzmann et al. [16]	2007	GER	23 M:11 F:12	46.7	Condylar inclination angle	Case series	NR	Ultrasonic tracking
Reichender et al. [17]	2013	GER	132 M:68 F:64	7.2 to 10.6	MMO CPIA	Cohort study	NR	Ultrasonic tracking
Schwestka-Polly et al. [18]	1999	GER	40 M:NR F:NR	NR	Mandibular rotation	Case control study	NR	Ultrasonic tracking
Stiesch-Scholz et al. [19]	2006	GER	60 M:NR F:NR	NR	Condylar trajectories	Case control study	NR	Ultrasonic tracking
Teng et al. [20]	2015	JAP	63 M:17 F:46	NR	Condylar and incisal trajectories	Cohort study	grant (CMRPG 381623) from Chang Gung Memorial Hospital	Ultrasonic tracking

Wang et al. [21]	2009	CHI	44 M:23 F:21	Test: 23.1 Control:22.5	Condylar trajectories	Cohort study	NR	Ultrasonic tracking
Yen at al. [22]	2017	CHI	20 M:18 F:2	Test: 28.9 ± 2.4 Control: 29.4 ± 3.2	Condylar trajectories	Cohort study	NR	Ultrasonic tracking
Baeyens et al. [23]	2013	CH	12 M:5 F:7	NR	Condylar and helical axis trajectories	Case series	NR	Magnetic Tracking
Baltali et al. [24]	2008	TUR	5 M:0 F:5	NR	Condylar and incisal trajectories	Case series	NR	Magnetic Tracking
Keller et al. [25]	2012	USA	36 M:2 F:34	46	Condylar, incisal and helical axis trajectories	Cohort study	NR	Magnetic Tracking
Minami et al. [26]	2018	JAP	14 M:6 F:8	NR	Incisal trajectories and mandibular rotation	Case series	Grant-in-Aid for Scientific Research from the Japan Society for the Promotion of Science	Magnetic Tracking
Yamamoto et al. [27]	2009	JAP	25 M:15 F:5	29.3 ± 12.3	Intraarticular distance	Case series	NR	Magnetic Tracking
Chang et al. [28]	2015	JAP	26 M:10 F:16	19 to 25	Intraarticular distance	Cohort study	grant (HI13C1491) from the Korea Health Technology R&D Project, Ministry of Health and Welfare, Republic of Korea	Optoelectronic tracking
Ettlin et al. [29]	2008	CH	10 M:5 F:5	18 to 31	Intraarticular distance, condilar tajectories	Case series	standard financial plan of the University of Zürich and by Grant of the Swiss National Science Foundation	Optoelectronic tracking
Fushima et al. [30]	2003	JAP	10 M:3 F:7	NR	Intraarticular distance	Case series	The standard financial	Optoelectronic tracking

							plan of the University of Zurich.	
Gallo et al. [31]	2008	CH	11 M:4 F:7	28 to 38	Intraarticular distance, condilar tajectories	Case series	standard financial plan of the University of Zürich and by Grant of the Swiss National Science Foundation	Optoelectronic tracking
Goto et al. [32]	2001	NED	4 M:4 F:0	30 to 35	Muscular lenght	Case series	MRC (Canada)*	Optoelectronic tracking
Hayasaki et al. [33]	2008	JAP	20 M:0 F:20	19 to 27	Mandibular rotation	Case series	Japanese society for the promotion of science	Optoelectronic tracking
Kim et al. [34]	2013	KOR sud	15 M:4 F:11	F: 21.4 M: 26.3	Condylar trajectories	Case series	Industrial Strategic Technology Development Program and the research Grant from the Seoul National University	Optoelectronic tracking
Kim et al. [35]	2010	KOR sud	16 M:6 F:8	17 to 27	Condylar trajectories	Case series	NR	Optoelectronic tracking
Kubota et al. [36]	2010	JAP	48 M:11 F:37	Test: 5.9 Control: 25.9	Condylar trajectories and cycle mastication data	Cohort study	Kakenhi from Japan society for the promotion of science	Optoelectronic tracking
Leader et al. [37]	2003	USA	6 M:3 F3:	20 to 47	Condylar trajectories	Case series	NIDCR grant from the National Institutes of Health, Bethesda, USA	Optoelectronic tracking
Wojczynska et al. [38]	2019	CH	5 M:1 F:4	22 to 61	Condylar trajectories	Case series	grant from the AO Foundation, Du¨bendorf, Switzerland	Optoelectronic tracking
Yashiro et al. [39]	2015	JAP	20 M:0 F:20	18 to 36	Condylar trajectories and intraarticular joint space	Case series	Grant-in-Aid for Scientific Research from the Japanese Ministry of Education, Science and Culture.	Optoelectronic tracking
Yashiro et al. [40]	2015	JAP	10 M.0 F:10	24 to 33	Condylar trajectorie	Case series	Grant-in-Aid for Scientific Research	Optoelectronic tracking

					s and intraarticular joint space		from the Japanese Ministry of Education, Science and Culture.	
Zaugg et al. [41]	2012	CH	6 M:4 F:2	54 to 71	Condylar trajectories and intraarticular joint space	Case series	standard financial plan of the University of Zurich	Optoelectronic tracking
Abbink et al. [42]	1999	NED	8 M:4 F:4	20 to 50	Mandibular traslation	Case series	Faculty of Medicine of the Utrecht University and the Netherlands Institute for Dental Sciences	3D scanning
Amhamed et al. [43]	2016	AUS	14 M:9 F:5	33.8 ± 3.8	Mandibular traslation	Case series	NR	3D scanning
Bhaskaracharya et al. [44]	2015	AUS	31 M:8 F:23	Test: 49 to 76 Control: 50 to 78	Incisal trajectories	Case control study	Australian Dental Research Foundation	3D scanning
Buschang et al. [45]	2001	USA	27 M:0 F:27	23 to 35	Condylar and incisal trajectories	Case series	Center for Craniofacial Research and Diagnosis, Baylor College of Dentistry,	3D scanning
Buschang et al. [46]	2000	USA	26 M:15 F:11	20 to 35	mastication cycle data	Cohort study	NR	3D scanning
Calixtre et al. [47]	2017	BRA	36 M:19 F:17	23.92 ± 2.8	Condylar trajectories	Case series	São Paulo Research Foundation	3D scanning
Catic, Naeije [48]	1999	CRO	40 M:13 F:27	Test: 16 to 61 Control: 21 to 33	Hinge axis	Case control study	NR	3D scanning
Coutant et al. [49]	2008	FRA	32	NR	Condylar trajectories	Case series	CNRS UMR 5227 Laboratoire Mouvement Adaptation Cognition” (CHIEDI)	3D scanning
Crane et al. [50]	2013	USA	22 M.2 F:20	10 to 58	Mandibular translatio	Case series	Grants awarded to GEG from the National Institutes of Health.	3D scanning

					n and velocity			
De felicio et al. [51]	2013	ITA	20 M:8 F: 12	Test: 27.3 ± 7.8 Control: 31.9 ± 16.3	Condylar and incisal trajectories, cycle mastication data	Case control study	Fundação de Amparo a Pesquisa do Estado de São Paulo, Brazil	3D scanning
Ferrario et al. [52]	2005	ITA	27: M:12 F:15	19 to 30	incisal trajectories	Case series	NR	3D scanning
Gallo et al. [53]	1997	CH	30 M:17 F:13	10 to 34	FHA fluctuation MMO	Case series	standard financial plan of the University of Zurich	3D scanning
Gallo et al. [54]	2000	CH	7 M:2 F:5	22 to 38	Helical axis trajectories	Case series	standard financial plan of the University of Zurich	3D scanning
Gallo et al. [55]	2006	CH	50 M:12 F:38	Test: 26 to 51 Control: 25 to 39	Finite Helical axis Fluctuation	Case control study	standard financial plan of the University of Zurich	3D scanning
Gerstner et al. [56]	2005	USA	46 M:13 F:33	19 to 62	mandibular trajectories	Case series	USPHS Research Grant from the National Institute of Dental Research.	3D scanning
Hayashi et al. [57]	2009	JAP	10 M:5 F:5	NR	Finite helical axis	Case series	NR	3D scanning
Hayashi et al. [58]	2012	JAP	10 M:7 F:3	25±7.0	Finite helical axis	Case series	Grant-in-Aid for Young Scientists from the Japan Society for the Promotion of Science	3D scanning
Leader et al. [59]	1997	USA	20 M:3 F:17	31.2 ± 11.2	Incisal trajectories	Case series	NIH grant	3D scanning
Lemoine et al. [60]	2005	USA	10 M:5 F:5	21 to 47	Mandibula centre of rotation	Case series	NR	3D scanning
Lewis et al. [61]	2001	USA	56 M:29 F:27	Test: 23 to 39 Control: 23 to 35	Kinematic hinge axis and incisal	Case control study	NR	3D scanning

					trajectories			
Lobezoo et al. [62]	2000	NED	5 M:5 F:0	20 to 25	Condylar traslation and rotation	Case series	Netherlands Institute for Dental Sciences (IOT)	3D scanning
Mapelli et al. [63]	2009	ITA	26 M:12 F:14	19 to 30	Condylar trajectories	Cohort study	NR	3D scanning
Mapelli et al. [64]	2016	ITA	40 M:5 F:35	Test: 18 to 34 Control: 20 to 31	Condylar and incisal trajectories	Case control study	Provost's Office for Research of the University of Sao Paulo, Brazil, and Nacional Council for Scientific and Technological Development	3D scanning
Naeije [65]	2002	NED	35 M:17 F:18	18 to 31	Condylar translation and rotation	Case series	NR	3D scanning
Naeije e Hofman [66]	2003	NED	10 M:5 F:5	21 to 33	Condylar trajectories	Case series	Netherlands Institute of Dental Sciences (IOT)	3D scanning
Ostry et al. [67]	1997	CAN	5 M:5 F:0	NR	Kinematic hinge axis	Case series	NIH grant from the National Institute on Deafness and Other Communication Disorders, USA; ATR Human Information Processing Research Laboratories, Japan, NSERC-Canada, FCAR, Quebec	3D scanning
Ogawa et al. [68]	1998	JAP	41 M:16 F:25	19 to 28	Incisal trajectories	Case series	NR	3D scanning
Remijn et al. [69]	2017	NED	22 M:9 F:13	Test: 17 to 31 Control: 28 to 32	Mandibular trajectories and mastication cycle data	Case control study	NR	3D scanning
Sforza et al. [70]	2009	ITA	9 M:8 F:1	18 to 58	Condylar translatio	Cohort study	Annual grant from Milan University	3D scanning

					n and rotation			
Sforza et al. [71]	2010	ITA	58 M:28 F:30	24	Kinematic hinge axis	Case control study	NR	3D scanning
Sforza et al. [72]	2011	ITA	46 M:23 F:23	18 to 50	Incisal trajectories	Case control study	NR	3D scanning
Shiller et al. [73]	1999	CAN	7 M:2 F:5	NR	Mandibular translation and rotation	Case series	NR	3D scanning
Shiller et al. [74]	2000	FRA	8	NR	Mandibular rotation	Case series	NR	3D scanning
Siegler et al. [75]	1991	USA	3	NR	Condylar trajectories	Case series	NR	3D scanning
Slater et al. [76]	1999	NED	10 M:4 F:6	20 to 32	Condylar trajectories	Case series	Netherlands Institute of Dental Sciences (IOT).	3D scanning
Tomonari et al. [77]	2017	JAP	32 M:22 F:10	19 to 25	Condylar trajectories	Case series	JSPS KAKENHI	3D scanning
Travers et al. [78]	2000	JAP	27 M:0 F:27	23 to 35	Condylar and incisal trajectories	Case series	Center for Craniofacial Research and Diagnosis, Baylor College of Dentistry, a Member of the Texas A&M University System Health Science Center	3D scanning
Ugolini et al. [79]	2017	ITA	18 M:6 F:12	Test: 24 to 48 Control: 21 to 45	Condylar and incisal trajectories	Case control study	NR	3D scanning
Ugolini et al. [80]	2018	ITA	31 M:12 F:19	Test:22 to 48 Control: 19 to 29	Mandibular translation and rotation	Case control study	NR	3D scanning
Visscher et al. [81]	2000	NED	10 M:4 F:6	20 to 32	Condylar trajectories	Case series	NR	3D scanning

Zhao et al. [82]	2012	AUS	26 M:12 F:14	Test: 29.5 ± 4.9 Control: 29.6 ± 3.4	Incisal trajectories and mastication cycle data	Case control study	Postgraduate Scholarship in Neuroscience of the University of Sidney and the National Healt and Medical Research Council of Australia	3D scanning
Dos Santos [83]	2019	BRA	12 M:6 F:6	22.2 ± 4.7	Condylar trajectories	Case series	NR	Ultrasonic tracking
Dos Santos [84]	2021	BRA	184 M:44 F: 140	18 to 45	Condylar trajectories	Cross sectional	Department of Postgraduate Program in Rehabilitation Sciences, Universidade Nove de Julho—UNINOVE.	Mechanical linkage system
Ernst et al. [85]	2019	GER	13 M:0 F:13	21 to 31	Mandibular rotation	Case series	Deutsche Forschungsgemeinschaft	Ultrasonic tracking
Chen et al. [86]	2020	TW	10 M:5 F:5	32.3 ± 8.5	Mandibular rotation	Cross sectional	NR	3d fluoroscopy
Leissner et al. [87]	2021	CHI	476 M:92 F:384	31.03 ± 15.8	Condylar trajectories	Case series	NR	Mechanical linkage system
Choi et al. [88]	2020	USA	23	12.8 to 16.7	Incisal trajectories	Cohort study	NR	
Feng et al. [89]	2021	CHINA	6 M:4 F:3	21.7 ± 1.25	Condylar trajectories	Case series	National Natural Science Foundation of China, Strategic cooperation project be- tween Sichuan University and Yibin municipal people's government and Fundamental Research Funds for the Central Universities of China.	Optoelectronic tracking
Shu et al. [90]	2020	CHINA	5 M:3 F:2	20.6 ± 2.4	Condylar trajectories	Case series	NR	Video motion analysis

Shu et al. [91]	2021	CHINA	13 M:10 F:3	21.8 ± 2.1	Condylar trajectorie s	Case series	National Natural Science Foundation of China, Strategic cooperation project between Sichuan University and Yibin municipal people's government, the Innovative Spark Project of Sichuan University, the Technology Innovation Project of Science and Technology Bureau of Chengdu, and Fundamental Research Funds for the Central Universities of China.	Optoelectronic tracking

References

1. Bernhardt, O.; Küppers, N.; Rosin, M.; Meyer, G. Comparative tests of arbitrary and kinematic transverse horizontal axis recordings of mandibular movements, *J. Prosthet. Dent.* **2003**, *89*, 175–179, doi: 10.1067/mpr.2003.10.
2. Ghodsi, S.; Rasaeipour, S. Revising average condylar inclinations using electronic pantograph assessment: a cross-sectional study. *Dent. Hypotheses.* **2018**, *9*, 84–89.
3. Hirai, S.; Ogawa, T.; Shigeta, Y.; Ando, E.; Hirabayashi, R.; Ikawa, T.; Kasama, S.; Fukushima, S.; Matsuka, Y. Characteristics of mandibular movements in patients with square mandible. *Oral Surg., Oral Med. Oral Pathol. Oral Radiol.Endodontol.* **2009**, *108*, e75–e81.
4. Hüe, O. Condylar paths during protrusion in edentulous patients: analysis with electronic axiography. *J. Prosthodont.* **2011**, *20*, 294–298.
5. Sadat-Khonsari, R.; Fenske, C.; Kahl-Nieke, B.; Kirsch, I.; Jüde, H.D. Mandibular instantaneous centers of rotation in patients with and without temporomandibular dysfunction. *J. Orofac. Orthop*. **2003**, *64*, 256–264.
6. Sadat-Khonsari, R.; Fenske, C.; Kahl-Nieke, B.; Kirsch, I.; Jüde, H.D. The helical axis of the mandible during the opening and closing movement of the mouth. *J. Orofac. Orthop*. **2003**, *64*, 178–185.
7. Tamaki, K.; Celar, A.G.; Beyrer, S.; Aoki, H. Reproduction of excursive tooth contact in an articulator with computerized axiography data. J. Prosthet. Dent.1997, 78, 373–378.
8. Wieckiewicz, M.; Zietek, M.; Nowakowska, D.; Wieckiewicz, W. Comparison of selected kinematic facebows applied to mandibular tracing. *Biomed. Res. Int.* **2014**, *2014*, 818694.
9. Baqaien, M.A.; Al-Salti, F.M.; Muessig, D. Changes in condylar path inclination during maximum protrusion between the ages of 6 and 12 years. *J. Oral Rehabil.* **2007**, *34*, 27–33.
10. Kiseri, B.; Dayan, Ç.; Yildiz, M.; Sülün, T. The correlation between direction and amount of retrusive movement and condyle position and joint space. *Cranio.* **2018**, *36*, 250–256.
11. Ko, E.W.-C.; Huang, C.S.; Lo, L.-J.; Chen, Y.-R. Longitudinal observation of mandibular motion pattern in patients with skeletal class III malocclusion subsequent to orthognathic surgery. *J. Oral Maxillofac. Surg.* **2012**, *70*, e158–e168.
12. Ko, E.W.-C.; Alazizi, A.I.; Lin, C.-H. Three-dimensional surgical changes of mandibular proximal segments affect outcome of jaw motion analysis. *J. Oral Maxillofac. Surg.* **2015**, *73*, 971–984.
13. Kordaß, B; Bernhardt, O.; Ratzmann, A.; Hugger, S.; Hugger, A. Standard and limit values of mandibular condylar and incisal movement capacity. *Int. J. Comput. Dent*. **2014**, *17*, 9–20.

14. Linsen, S.S.; Wolf, M.; Mussig, D. Long-term outcomes of mandibular kinematics following class II malocclusion therapy with removable functional appliance therapy. *Cranio.* **2016**, *34*, 363–370.
15. Linsen, S.S.; Schön, A.; Mercuri, L.G.; Teschke, M.; Share. Unilateral, Alloplastic Temporomandibular Joint Reconstruction, Biomechanically What Happens to the Contralateral Temporomandibular Joint?-A Prospective Cohort Study. *J Oral Maxillofac Surg.* **2021**, *10*, 2016-2029.
16. Ratzmann, A.; Mundt, T.; Schwahn, C.; Langforth, G.; Hützen, D.; Gedrange, T.; Kordaß, B. Comparative clinical investigation of horizontal condylar inclination using the JMA electronic recording system and a protrusive wax record for setting articulators. *Int. J. Comput. Dent.* **2007**, *10*, 265–284.
17. Reicheneder, C.; Kardari, Z.; Proff, P.; Fanghaenel, J.; Faltermeier, A.; Römer, P. Correlation of condylar kinematics in children with gender, facial type and weight. *Ann. Anat.* **2013**, *195*, 243–247.
18. Schwestka-Polly, R.; Kubein-Meesenburg, D.; Nagerl, H.; Fanghanel, J.; Miehe, B. Alteration of the functional condition of the mandible during clinical treatment. *Ann. Anat.* **1999**, *181*, 45–50.
19. Stiesch-Scholz, M.; Demling, A.; Rossbach, A. Reproducibility of jaw movements in patients with craniomandibular disorders. *J. Oral Rehabil.* **2006**, *33*,807–812.
20. Teng, T.T.-Y.; Ko, E.W.-C.; Huang, C.S.; Chen, Y.-R. The Effect of early physiotherapy on the recovery of mandibular function after orthognathic surgery for Class III correction: Part I - Jaw-motion analysis. *J. Cranio-Maxillofacial Surg.* **2015**, *43*, 131–137.
21. Wang, D.; Fu, H.; Zeng, R.; Yang, X. Changes of mandibular movement tracings after the correction of mandibular protrusion by bilateral sagittal split ramus osteotomy. *J. Oral Maxillofac. Surg.* **2009**, *67*, 2238–2244.
22. Yen, C.-I.; Chou, P.-Y.; Chen, C.-H.; Chen, T.-Y.; Chen, C.-T.; Lin, W.-Y.; Lee, M.-Y. Kinematic, Kinetic and surface electromyography analysis following zygomatic fracture reconstruction. *J. Med. Biol. Eng.* **2017**, *37*, 468–473.
23. Baeyens, J.P.; Gilomen, H.; Erdmann, B.; Clijsen, R.; Cabri, J.; Vissers, D. In vivo measurement of the 3D kinematics of the temporomandibular joint using miniaturized electromagnetic trackers: technical report. *Med. Biol. Eng. Comput.* **2013**, *51*, 479–484.
24. Baltali, E.; Zhao, K.D.; Koff, M.F.; Durmus, E.; An, K.-N.; Keller, E.E. A method for quantifying condylar motion in patients with osteoarthritis using an electromagnetic tracking device and computed tomography imaging. *J. OralMaxillofac. Surg.* **2008**, *66*, 848–857.
25. Keller, E.E.; Baltali, E.; Liang, X.; Zhao, K.; Huebner, M.; An, K.-N. Temporomandibular custom hemijoint replacement prosthesis: prospective clinical and kinematic study. *J. Oral Maxillofac. Surg.* ***2012***, *70*, 276–288.
26. Minami, I.; Wirianski, A.; Harakawa, R.; Wakabayashi, N.; Murray, G.M. The three-axial gyroscope sensor detects the turning point between opening and closing phases of chewing. *Clin. Exp. Dent. Res.* **2018**, *4*, 249–254.
27. Yamamoto, T.; Nishigawa, K.; Bando, E.; Hosoki, M. Effect of different head positions on the jaw closing point during tapping movements. *J. Oral Rehabil.* **2009**, *36*, 32–38.
28. Chang, A.R.; Han, J.J.; Kim, D.-S.; Yi, W.-J.; Hwang, S.J. Evaluation of intraarticular distance narrowing during tem-poromandibular joint movement in patients with facial asymmetry using 3-dimensional computed tomography image and tracking camera system. *J. Cranio-Maxillofacial Surg.* **2015**, *43*, 342–348.
29. Ettlin, D.A.; Mang, H.; Colombo, V.; Palla, S.; Gallo, L.M. Stereometric assessment of TMJ space variation by occlusal splints. *J. Dent. Res.* **2008**, *87*, 877–881.
30. Fushima, K.; Gallo, L.M.; Krebs, M.; Palla, S. Analysis of the TMJ intraarticular space variation: a non-invasive insight during mastication. *Med. Eng. Phys.* **2003**, *25*, 181–190.
31. Gallo, L.M.; Gössi, D.B.; Colombo, V.; Palla, S. Relationship between kinematic center and TMJ anatomy and function. *J. Dent. Res.* **2008**, *87*, 726–730.
32. Goto, T.K.; Langenbach, G.E.J.; Hannam, A.G. Length changes in the human masseter muscle after jaw movement. *Anat. Rec.* **2001**, *262*, 293–300.
33. Hayasaki, H.; Saitoh, I.; Iwase, Y.; Inada, E.; Hasegawa, H.; Tokutomi, J.; Matsumoto,Y.; Yamasaki, Y. Movement of the instantaneous center of rotation and the position of the lateral excursion center during lateral excursion. *Cranio - J. Crani-omandib. Pract.* **2008**, *26*, 253–262.
34. Kim, D.-S.; Choi, S.-C.; Lee, S.-S.; Heo, M.-S.; Huh, K.-H.; Hwang, S.-J.; Kim, S.-H.; Yi, W.-J. Principal direction of inertia for 3D trajectories from patientspecific TMJ movement. *Comput. Biol. Med.* **2013**, *43*, 169–175.
35. Kim, S.-G.; Kim, D.-S.; Choi, S.-C.; Lee, S.-S.; Heo, M.-S.; Huh, K.-H.; Hwang, S.-J.; Yi,W.-J. The relationship between three-dimensional principal rotations and mandibular deviation. *Oral Surg., Oral Med. Oral Pathol. Oral Radiol.Endodontol.* **2010**, *110*, 52–60.
36. Kubota, N.; Hayasaki, H.; Saitoh, I.; Iwase, Y.; Maruyama, T.; Inada, E.; Hasegawa, H.; Yamada, C.; Takemoto, Y.; Matsumoto, Y.; Yamasaki, Y. Jaw motion during gum-chewing in children with primary dentition. *Cranio-J. Craniomandib.Pract.* **2010**, *28*, 19–29.
37. Leader, J.K.; Boston, J.R.; Rudy, T.E.; Greco, C.M.; Zaki, H.S. Relation of jaw sounds and kinematics visualized and quantified using 3-D computer animation. *Med. Eng. Phys.* **2003**, *25*, 191–200.

38. Wojczynska, A.; Gallo, L.M.; Bredell, M.; Leiggener, C.S. Alterations of mandibular movement patterns after total joint replacement: a case series of long-term outcomes in patients with total alloplastic temporomandibular joint reconstructions. *Int. J. Oral Maxillofac. Surg.* **2019**, *48*, 225–232.
39. Yashiro, K.; Iwata, A.; Takada, K.; Murakami, S.; Uchiyama, Y.; Furukawa, S. Temporomandibular joint articulations on working side during chewing in adult females with cross-bite and mandibular asymmetry. *J. Oral Rehabil.* **2015**, *42*,163–172.
40. Yashiro, K.; Yamamoto, K.; Takada, K.; Murakami, S.; Uchiyama, Y.; Furukawa, S. Influence of balancing-side occlusal interference on smoothness of working-side condylar movement and intra-articular space in chewing efforts. *J. Oral Rehabil.* **2015**, *42*, 10–17.
41. Zaugg, B.; Hämmerle, C.H.F.; Palla, S.; Gallo, L.M. Implant-supported mandibular splinting affects temporomandibular joint biomechanics. *Clin.Oral Implants Res.* **2012**, *23*, 897–901.
42. Abbink, J.H.; Van Der Bilt, A.; Bosman, F.; Van Der Glas, H.W. Speed-dependent control of cyclic open-close movements of the human jaw with an externalforce counteracting closing. *J. Dent. Res.* **1999**, *78*, 878–886.
43. Amhamed, M.; Whittle, T.; Maulina, T.; Gal, J.; Akhter, R.; Murray, G.M. Effect of experimental anterior temporalis muscle pain on jaw movements. *J. Oral Rehabil.* **2016**, *43*, 889–899.
44. Bhaskaracharya, M.; Memon, S.M.; Whittle, T.; Murray, G.M. Jaw movements in patients with a history of pain: an exploratory study. *J. Oral Rehabil.* **2015**, *42*, 18–26.
45. Buschang, P.H.; Throckmorton, G.S.; Travers, K.H.; Hayasaki, H. Incisor and mandibular condylar movements of young adult females during maximum protrusion and lateratrusion of the jaw. *Arch. Oral Biol.* **2001**, *46*, 39–48.
46. Buschang, P.H.; Hayasaki, H.; Throckmorton, G.S. Quantification of human chewing-cycle kinematics. *Arch. Oral Biol.* **2000**, *45*, 461–474.
47. Calixtre, L.B.; Nakagawa, T.H.; Alburquerque-Sendin, F.; da Silva Gruninger, B.L.; de Sena Rosa, L.R.; Oliveira, A.B. Inter- and intra-rater reliability of 3D kinematics during maximum mouth opening of asymptomatic subjects. *J.Biomech.* **2017**, *64*, 245–252.
48. C′atic, A.; Naeije, M. Location of the hinge axis and the kinematic centre in asymptomatic and clicking temporo-mandibular joints. *J. Oral Rehabil.* **1999**, *26*, 661–665.
49. Coutant, J.-C.; Mesnard, M.; Morlier, J.; Ballu, A.; Cid, M. Discrimination of objective kinematic characters in temporomandibular joint displacements. *Arch. Oral Biol.* **2008**, *53*, 453–461.
50. Crane, E.A.; Rothman, E.D.; Childers, D.; Gerstner, G.E. Analysis of temporal variation in human masticatory cycles during gum chewing. *Arch. Oral Biol.* **2013**, *58*, 1464–1474.
51. De Felicio, C.M.; Mapelli, A.; Sidequersky, F.V.; Tartaglia, G.M.; Sforza, C. Mandibular kinematics and masticatory muscles EMG in patients with short lasting TMD of mild-moderate severity. *J. Electromyogr. Kinesiol.* **2013**, *23*, 627–633.
52. Ferrario, V.F.; Sforza, C.; Lovecchio, N.; Mian, F. Quantification of translational and gliding components in human temporomandibular joint during mouth opening. *Arch. Oral Biol*. **2005**, *50*, 507–515.
53. Gallo, L.M.; Airoldi, G.B.; Airoldi, R.L.; Palla, S. Description of mandibular finite helical axis pathways in asymptomatic subjects. *J. Dent. Res*. **1997**, *76*, 704–713.
54. Gallo, L.M.; Fushima, K.; Palla, S. Mandibular helical axis pathways during mastication. *J. Dent. Res.* **2000**, *79*, 1566–1572.
55. Gallo, L.M.; Brasi, M.; Ernst, B.; Palla, S. Relevance of mandibular helical axis analysis in functional and dysfunctional TMJs. *J. Biomech.* **2006**, *39*, 1716–1725.
56. Gerstner, G.E.; Lafia, C.; Lin, D. Predicting masticatory jaw movements from chin movements using multivariate linear methods. *J. Biomech.* **2005**, *38*, 1991–1999.
57. Hayashi, K.; Reich, B.; Delong, R.; Lee, S.-P.; Mizoguchi, I. A novel statistical model for mandibular helical axis analysis. J. Oral Rehabil. **2009**, *36*, 102–109.
58. Hayashi, K.; Hayashi, M.; Reich, B.; Lee, S.-P.; Sachdeva, A.U.C.; Mizoguchi, I. Functional data analysis of mandibular movement using third-degree b-spline basis functions and self-modeling regression. *Orthod. Waves.* **2012**, *71*, 17–25.
59. Leader, J.K.; Boston, J.R.; Rudy, T.E.; Greco, C.M.; Zaki, H.S. Use of sound and jaw movement patterns to characterize patients with temporomandibulardisorders. *Annu. Int. Conf IEEE Eng. Med. Biol. - Proc.* **1997**, *4*, 1729–1732.
60. Lemoine, J.J.; Xia, J.J.; Gateno, J.; Liebschner, M.A.K. Radiographic analysis for jaw motion normalization. *J. Oral Maxillofac. Surg.* **2005**, *63*, 961–967.
61. Lewis, R.P.; Buschang, P.H.; Throckmorton, G.S. Sex differences in mandibular movements during opening and closing. Am. J. Orthod. Dentofac. Orthop. **2001**, *120*, 294–303.
62. Lobbezoo, F.; Zwijnenburg, A.J.; Naeije, M. Functional subdivision of the human masseter and temporalis muscles as shown by the condylar movement response to electrical muscle stimulation. *J. Oral Rehabil.* **2000**, *27*, 887–892.
63. Mapelli, A.; Galante, D.; Lovecchio, N.; Sforza, C.; Ferrario, V.F. Translation and rotation movements of the mandible during mouth opening and closing. *Clin. Anat*. **2009**, *22*, 311–318.
64. Mapelli, A.; Machado, B.C.Z.; Garcia, D.M.; Rodrigues Da Silva, M.A.M.; Sforza, C.; DeFelicio, C.M. Three-dimensional analysis of jaw kinematic alterations in patients with chronic TMD—Disc displacement with reduction. *J. Oral Rehabil.* **2016**, *43*, 824–832.
65. Naeije, M. Local kinematic and anthropometric factors related to the maximum mouth opening in healthy individuals. *J. Oral Rehabil.* **2002**, *29*, 534–539.

66. Naeije, M.; Hofman, N. Biomechanics of the human temporomandibular joint during chewing. *J. Dent. Res.* **2003**, *82*, 528–531.
67. Ostry, D.J.; Vatikiotis-Bateson, E.; Gribble, P.L. An examination of the degrees of freedom of human jaw motion in speech and mastication. *J. Speech Lang. Hear. Res.* **1997**, *40*, 1341–1351.
68. Ogawa, T.; Koyano, K.; Umemoto, G. Inclination of the occlusal plane and occlusal guidance as contributing factors in mastication. *J. Dent.* **1998**, *26*, 641–647
69. Remijn, L.; Groen, B.E.; Speyer, R.; van Limbeek, J.; Vermaire, J.A.; van den Engel-Hoek, L.; Nijhuis-van der Sanden, M.W.G. Can mastication in children with cerebral palsy be analyzed by clinical observation, dynamic ultrasound and 3D kinematics? *J. Electromyogr. Kinesiol.* **2017**, *32*, 22–29.
70. Sforza, C.; Tartaglia, G.M.; Lovecchio, N.; Ugolini, A.; Monteverdi, R.; Giannì, A.B.; Ferrario, V.F. Mandibular movements at maximum mouth opening and EMG activity of masticatory and neck muscles in patients rehabilitated after a mandibular condyle fracture. *J. Cranio-Maxillofacial Surg.* **2009**, *37*, 327–333.
71. Sforza, C.; Ugolini, A.; Rocchetta, D.; Galante, D.; Mapelli, A.; Giannì, A.B.Mandibular kinematics after orthognathic surgical treatment. A pilot study. *Br.J. Oral Maxillofac. Surg.* **2010**, *48*, 110–114.
72. Sforza, C.; Ugolini, A.; Sozzi, D.; Galante, D.; Mapelli, A.; Bozzetti, A. Threedimensional mandibular motion after closed and open reduction of unilateral mandibular condylar process fractures. *J. Cranio-Maxillofacial Surg.* **2011**, *39*, 249–255.
73. Shiller, D.M.; Ostry, D.J.; Gribble, P.L. Effects of gravitational load on jaw movements in speech. *J. Neurosci.* **1999**, *19*, 9073–9080.
74. Shiller, D.M.; Ostry, D.J.; Gribble, P.L.; Laboissière, R. Compensation for the effects of head acceleration on jaw movement in speech. *J. Neurosci.* **2001**, *21*, 6447–6456.
75. Siegler, S.; Hayes, R.; Nicolella, D.; Fielding, A. A technique to investigate the three-dimensional kinesiology of the human temporomandibular joint. *J. Prosthet. Dent.* **1991**, *65*, 833–839.
76. Slater, J.; Visscher, C.M.; Lobbezoo, F.; Naeije, M. The intra-articular distance within the TMJ during free and loaded closing movements. *J. Dent. Res.* **1999**, *78*, 1815–1820.
77. Tomonari, H.; Kwon, S.; Kuninori, T.; Miyawaki, S. Differences between the chewing and non-chewing sides of the mandibular first molars and condyles in the closing phase during chewing in normal subjects. *Arch. Oral Biol.* **2017**, *81*, 198–205.
78. Travers, K.H.; Buschang, P.H.; Hayasaki, H.; Throckmorton, G.S. Associations between incisor and mandibular condylar movements during maximum mouth opening in humans. *Arch. Oral Biol.* **2000**, *45*, 267–275.
79. Ugolini, A.; Mapelli, A.; Segu, M.; Galante, D.; Sidequersky, F.V.; Sforza, C. Kinematic analysis of mandibular motion before and after orthognathic surgery for skeletal Class III malocclusion: a pilot study. *Cranio*. **2017**, 35, 94–100.
80. Ugolini, A.; Mapelli, A.; Segu, M.; Zago, M.; Codari, M.; Sforza, C. Three dimensional mandibular motion in skeletal Class III patients. *CRANIO-J. Craniomandib. Pract.* **2018**, *36*, 113–120.
81. Visscher, C.M.; Huddleston Slater, J.J.R.; Lobbezoo, F.; Naeije, M. Kinematics of the human mandible for different head postures. *J. Oral Rehabil.* **2000**, *27*, 299–305.
82. Zhao, N.N.; Whittle, T.; Murray, G.M.; Peck, C.C. The effects of capsaicin induced intraoral mucosal pain on jaw movements in humans. *J. Oral Facial Pain Headache*. **2012**, *26*, 277–287.
83. Dos Santos, D.M.; Lucareli, P.R.; Borges Barbosa, B.R.; Alves de Azevedo L.M.; Leonardis, M.N.; Fonseca F.F.; de Carvalho, L.S.; Gonzalez,T.D.O.; Politti F.; Biasotto-Gonzalez, D.A. Reproducibility of a three-dimensional jaw model during opening and lateral excursion tests. *Gait Posture.* **2019**, *73*, 529–530.
84. Dos Santos, D.M.; Politti, F.; de Azevedo, L.M.A.; de Cassia das Neves Martins, R.; Ricci, F.C.; Masuda, K.S.Y.; Muramoto do Nascimento, E.M.; Spinato, I.L.; de Paula Gomes, C.A.F.; Biasotto-Gonzalez, D.A. Association between convergence insufficiency and temporomandibular disorder cross-sectional study. *Clin. Oral Investigations.* **2021**, *25*, 851–858.
85. Ernst, M.; Schenkenberger, A.E.; Domin, M.; Kordass, B.; Lotze, M. Effects of centric mandibular splint therapy on orofacial pain and cerebral activation patterns. *Clin. Oral Investigations.* **2020**, *24*, 2005–2013.
86. Chen, C.-C.; Lin, C.-C.; Hsieh, H.-P.; Fu, Y.-C.; Chen, Y.-J.; Lu, T.-W. In vivo three-dimensional mandibular kinematics and functional point trajectories during temporomandibular activities using 3d fluoroscopy. *Dentomaxillofacial Radiol*. **2021**, *50*, 20190464, doi: 10.1259/dmfr.20190464.
87. Leissner, O.; Maulén-Yáñez, M.; Meeder-Bella, W.; León-Morales, C.; Vergara-Bruna, E.; González-Arriagada, W.A. Assessment of mandibular kinematics values and its relevance for the diagnosis of temporomandibular joint disorders. *J. Dent. Sci.* **2021**, *16*, 241–248.
88. Choi, D.D.; Vandenberg, K.; Smith, D.; Davis, C.; McCain, J.P. Is Temporomandibular Joint Arthroscopy Effective in Managing Pediatric Temporomandibular Joint Disorders in the Short- and Long-Term? *J Oral Maxillofac Surg*. **2020**, *78*, 44–51
89. Feng, Y.; Shu, J.; Liu, Y.; Zheng, T.; Shao, B.; Liu, Z. Biomechanical analysis of temporomandibular joints during mandibular pro-trusion and retraction motions: A 3d finite element simulation. *Comput. Methods Programs Biomed.* **2021**, *208*, 106299.
90. Shu, J.; Ma, H.; Jia, L.; Fang, H.; Chong, D.Y.R.; Zheng, T.; Yao, J.; Liu, Z. Biomechanical behaviour of temporomandibular joints during opening and closing of the mouth: A 3D finite element analysis. *Int J Numer. Method Biomed Eng*. **2020**, *8*, e3373
91. Shu, J.; Ma, H.; Xiong, X.; Shao, B.; Zheng, T.; Liu, Y.; Liu, Z. Mathematical analysis of the condylar trajectories in asymptomatic subjects during mandibular motions. *Med Biol Eng Comput.* **2021**, *59*, 901–911.
